# Notes and Notices

**Published:** 1890-08

**Authors:** 


### NOTES AND NOTICES.

Removals—Dr. Charles P. Beaman, formerly located in Chicago, where he made a specialty of the treatment of the throat, heart, and lungs, has removed to Chattanooga, Tenn., where he will resume the *general* practice of medicine. Dr. C. H. Oakes has removed from Northboro' to Dighton, Mass. Dr. Wm. S. Gee, associate editor of *The Medical Advance*, is sojourning for his health at Manitou, Colorado. Dr. Fourness Simmons has removed from 4 Byrne Terrace, Wickham Terrace, to 5 Union Terrace, George Street, Brisbane, Queensland, Australia.

